# Parent–Child Eye Gaze Congruency to Emotional Expressions Mediated by Child Aesthetic Sensitivity

**DOI:** 10.3390/children12070839

**Published:** 2025-06-25

**Authors:** Antonios I. Christou, Kostas Fanti, Ioannis Mavrommatis, Georgia Soursou

**Affiliations:** 1 Department of Special Education, University of Thessaly, 38221 Volos, Greece; 2 Department of Psychology, University of Cyprus, 1678 Nicosia, Cyprus; kfanti@ucy.ac.cy (K.F.); mavrommatis.ioannis@ucy.ac.cy (I.M.); soursou.georgia@ucy.ac.cy (G.S.)

**Keywords:** sensory processing sensitivity, aesthetic sensitivity, empathy, eye gaze, emotion processing, parent–child dyads

## Abstract

Background/Objectives: Sensory Processing Sensitivity (SPS), particularly its aesthetic subcomponent (Aesthetic Sensitivity; AES), has been linked to individual differences in emotional processing. This study examined whether parental visual attention to emotional facial expressions predicts corresponding attentional patterns in their children, and whether this intergenerational concordance is mediated by child AES and moderated by child empathy. Methods: A sample of 124 Greek Cypriot parent–child dyads (children aged 7–12 years) participated in an eye-tracking experiment. Both parents and children viewed static emotional facial expressions (angry, sad, fearful, happy). Parents also completed questionnaires assessing their child’s SPS, empathy (cognitive and affective), and emotional functioning. Regression analyses and moderated mediation models were employed to explore associations between parental and child gaze patterns. Results: Children’s fixation on angry eyes was significantly predicted by parental fixation duration on the same region, as well as by child AES and empathy levels. Moderated mediation analyses revealed that the association between parent and child gaze to angry eyes was significantly mediated by child AES. However, neither cognitive nor affective empathy significantly moderated this mediation effect. Conclusions: Findings suggest that child AES plays a key mediating role in the intergenerational transmission of attentional biases to emotional stimuli. While empathy was independently associated with children’s gaze behavior, it did not moderate the AES-mediated pathway. These results highlight the importance of trait-level child sensitivity in shaping shared emotional attention patterns within families.

## 1. Introduction

Understanding how children process emotional stimuli in their environment—particularly within parent–child relationships—is central to developmental psychopathology. A growing body of research highlights the role of parental influences in shaping children’s emotional and cognitive development, including attention to emotional cues [1,2]. Yet the mechanisms through which these intergenerational patterns of emotion processing emerge remain unclear. Within this context, Environmental Sensitivity (ES) has gained traction as a meta-framework describing how individuals differ in their reactivity to both positive and negative environments [3,4]. A key trait within this framework is Sensory Processing Sensitivity (SPS)—a biologically based disposition characterized by increased emotional responsiveness, heightened perceptual awareness, and depth of processing [5,6].

Recent evidence suggests that SPS interacts with environmental inputs, such as parenting behaviors, to predict child outcomes, including externalizing and internalizing symptoms [7,8,9]. However, much of this work has focused on behavioral outcomes rather than neurocognitive mechanisms like attention to emotion, e.g., [10,11,12]. Moreover, the directionality of influence—whether parents shape child attention, or children’s traits modulate their response to parental cues—has been insufficiently explored. This study addresses that gap by examining whether children’s SPS statistically accounts for the association between parental and child eye gaze patterns in response to emotional facial expressions.

SPS is composed of three subdimensions: Ease of Excitation (EOE), Low Sensory Threshold (LST), and Aesthetic Sensitivity (AES) [13,14]. While EOE and LST are linked to overstimulation and behavioral inhibition (associated with the BIS system; [15]), AES—reflecting heightened awareness of subtleties, aesthetic appreciation, and sensitivity to emotional tone—is associated with the BAS system and traits such as empathy, creativity, and positive social engagement [16,17]. Despite these distinctions, few studies have investigated how specific SPS facets differentially relate to attention toward emotional information in children, particularly in the context of parent–child interactions.

A further dimension relevant to this dynamic is empathy, which shares conceptual overlap with SPS and has been positively associated with emotional reactivity and social awareness [18,19]. While empathy is often treated as a single construct, cognitive and affective components may exert distinct influences on how children process others’ emotions. For example, cognitive empathy may support understanding others’ emotions without emotional over-involvement, whereas affective empathy entails vicarious emotional engagement. The interplay between SPS and empathy may shape how children attend to emotionally salient cues from parents.

Previous theoretical models (e.g., [20]) propose that parent–child similarities in emotional information processing may reflect shared genetic and environmental influences. Empirical studies have shown that parents’ threat-related biases and emotional reactivity can shape similar patterns in their children [21]. However, these studies rarely test the mechanistic pathways through which child-level traits like SPS or empathy contribute to such transmission. Moreover, eye gaze—a sensitive and objective index of attention to emotion—is often overlooked in these intergenerational models.

To address these gaps, the current study examines how parental eye gaze toward emotional faces is associated with children’s corresponding gaze, and whether this association is statistically accounted for by children’s Aesthetic Sensitivity and varies as a function of their empathy. By using the same eye-tracking paradigm in both parents and children, we aimed to identify shared attentional patterns and explore the role of child-specific social-emotional traits.

Visual scanning patterns of children and one parent, usually the mother, were measured with an eye-tracker during the computerized presentation of static positive and negative emotional faces (i.e., angry, sad, fearful, happy; see [8]). Parental and child SPS and its subscales were also measured to account for the contribution of individual differences in environmental sensitivity in child-to-parent associations of emotional process. Additional social-emotional characteristics of children were also measured using parental reports. We hypothesized that:

1. Children and parents would exhibit similar eye gaze patterns when viewing emotional faces.
2. These patterns were expected to be statistically accounted for by children’s SPS, with a particular focus on AES.
3. Different SPS facets would be differentially associated with gaze patterns, such that higher AES would relate to greater attention to emotional cues (particularly positive emotions), while higher EOE-LST would relate to an increased gaze toward negative emotional cues.
4. Empathy (cognitive and affective) would moderate the mediating role of AES, enhancing or attenuating its effect on gaze synchrony with parents.

By testing these pathways in a well-characterized sample of middle-childhood dyads, this study seeks to advance our understanding of intergenerational emotional processing and the moderating role of children’s individual sensitivity profiles.

## 2. Materials and Methods

### 2.1. Participants

The sample consisted of 124 dyads of children paired with one of their biological parents (children’s mean age = 9.98 years, SD = 1.30; 48.8% female; parents’ mean age = 40.9 years, SD = 4.85; 87.7% female). Families were recruited from a database of a nationwide study involving 2141 Greek Cypriot elementary school students who had consented to be re-contacted for future research conducted by our laboratory. Inclusion criteria required children to be between 7 and 12 years old, have normal or corrected-to-normal vision, and no history of neurological or psychiatric diagnoses. Of the 200 families originally approached, 21 were excluded based on health-related criteria, and 39 either declined participation or did not complete the eye-tracking session. This resulted in 140 eligible and consenting families. Of these, 16 dyads were subsequently excluded due to technical issues during data collection (e.g., calibration failure, excessive movement, or incomplete recordings), resulting in a final sample of 124 parent–child dyads. All participants had normal or corrected-to-normal vision. Families were invited to participate based on a database compiled from nationwide studies conducted by our lab at the local University. The broader study encompassed 47 private and public preschools and 69 primary schools across three districts in Cyprus—Nicosia, Larnaca, and Limassol. The schools were randomly selected and provided with detailed information about the study’s aims and scope. Following approval from the relevant school boards, the families received a detailed description of the study. Each child, accompanied by one parent, visited the Developmental Psychopathology Lab, where the child completed an eye-tracking task focused on facial processing. The study was approved by the National Bioethics Committee (Approval No. 2019/73). Written informed consent was obtained from parents for both their own and their child’s participation. During the visit, the researcher explained the purpose of the study and invited the families to take part in the experiment. The parents also completed a set of online questionnaires via REDCap, a secure web-based platform, which assessed the child’s sociodemographic background and emotional-behavioral characteristics. Information regarding any history of epilepsy or other significant mental or physical conditions that might affect participation was also collected; no such history was reported. All participants were Greek Cypriots, the largest ethnolinguistic group in Cyprus, and Greek was their native language.

### 2.2. Assessment of Parental Behavior

The SPS instruments for both parents and children were translated into Greek, then back-translated into English, and adapted in accordance with the guidelines provided by the original standardization team [13,14].

Highly Sensitive Person Scale: Environmental sensitivity in adults was assessed using the 12-item short form of the Highly Sensitive Person (HSP) scale, a validated version of the original 27-item measure that retains similar psychometric and construct validity [22]. Participants rated each item on a 7-point Likert scale ranging from 1 (‘strongly disagree’) to 7 (‘strongly agree’). A total sensitivity score was calculated by averaging responses across all items, with higher scores indicating greater sensitivity. Three dimensions were included in this scale: LST (three items, e.g., “Are you made uncomfortable by loud noises?”), EOE (five items; e.g., “Do you get rattled when you have a lot to do in a short amount of time?”), AES (four items; e.g., “Do you seem to be aware of subtleties in your environment?”). The scale and its sub-dimensions had good internal consistency (Cronbach’s α = 0.80 for the whole scale; 0.79 for EOE; 0.75 for LST; and 0.77 for AES). Thus, mean scores of the total scale and its sub-dimensions were computed.

### 2.3. Assessment of Child Behavior

Child’s Sensory Processing Sensitivity: The Highly Sensitive Child (HSC) Scale, consisting of 21 items, was used to assess children’s Sensory Processing Sensitivity (SPS) based on parent ratings. Items were rated on a 7-point Likert scale ranging from 1 (‘not at all’) to 7 (‘extremely’; [14]). The scale comprises two dimensions: EOE-LST (Ease of Excitation/Low Sensory Threshold; 13 items; e.g., “My child gets nervous when he/she has to do a lot in little time”; Cronbach’s α = 0.61) and AES (Aesthetic Sensitivity; 8 items; e.g., “Some music can make my child really happy”; Cronbach’s α = 0.61). An overall SPS score was calculated as the mean of all 21 items (Cronbach’s α = 0.80). Internal consistency was interpreted as follows: α < 0.60 = low; 0.60–0.70 = acceptable; ≥ 0.70 = good [14]. In this study, only parent-reported SPS scores were included (mean parental age = 40.1 years; 121 females), as this was deemed the most appropriate approach based on previous findings of partial metric and scalar measurement invariance in child self-reports across gender, age, and country [22,23]. These variations suggest that children with similar environmental sensitivity may receive differing scores on the HSC scale. Notably, earlier research has found significant correlations between mother and child reports on the overall SPS score and the EOE-LST dimension, but not on the AES subscale [14]. To this end, taking the age range of the participants of our sample parental reports of SPS was deemed the most reliable approach.

Empathy: The Basic Empathy Scale—BES was employed to assess children’s cognitive and affective empathy [24]. The 20-item measure assesses the degree to which each person understands and feels the emotions of others. In particular, the cognitive component of empathy included items such as, ‘S/he is not usually aware of he/his loved one’s feelings’. The affective component of empathy included items such as, ‘S/he doesn’t become sad when he/she sees other people crying,’. For the cognitive component, Cronbach’s α was 0.61, and for the affective component, the alpha was 0.78, which was comparable to previous work [24]. Parents responded to each item on a five-point Likert-type scale.

Affective Problems: Child affective symptoms [i.e., anxiety (α = 0.81; 8 items) and depression (α = 0.86; 8 items)] were assessed with the Checkmate plus Child Symptom Inventory for Parents-4 (CSI-4; [25]). Example items for the depression subscale included ‘Seems unhappy or sad much of the time’ and ‘Withdraws from friends and family more than usual’. The anxiety subscale included items such as ‘Avoids certain situations or places because of fear or anxiety’ and ‘Is easily startled or seems on edge much of the time’. Previous research has provided evidence for the validity of the parent-reported symptoms measured with the CSI-4 in community and clinical samples in Cyprus and the U.S. [26,27].

### 2.4. Child Attentional Patterns

**Figure 1:**
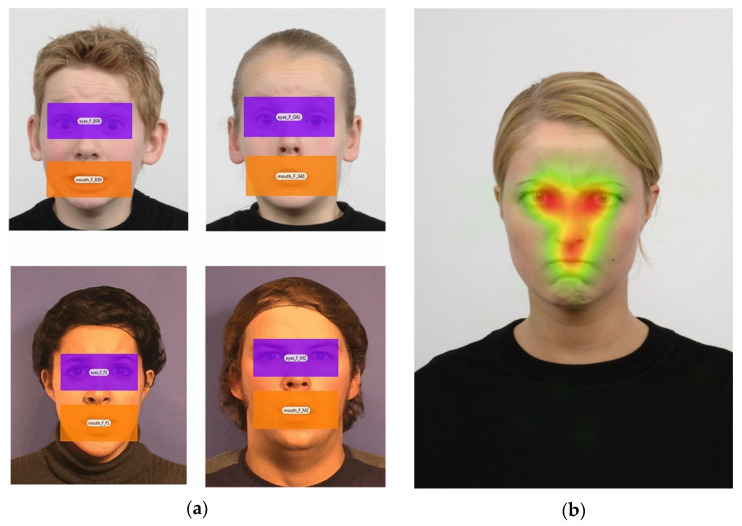
(**a**) Areas of interest (AOIs) used in the emotional processing eye-tracking task—examples from the expression of fear in male/female and child/adult actors. (**b**) A representative heatmap of gaze fixations during the viewing of an angry facial expression. Facial stimuli are drawn from the Radboud Faces Database [28], a publicly available and ethically approved dataset widely used in emotion research.

Eye-Tracking Experiment: The eye-tracking task consisted of static, front-facing images of adult and child actors displaying four emotional expressions: anger, sadness, fear, and happiness. Stimuli were sourced from the Radboud Faces Database [28] and had been previously validated in studies involving Cypriot children [8,9,29]. Specifically, the stimuli included images of four adult actors (50% female), each displaying four emotions across two snapshots (4 actors × 4 emotions × 2 images), and four child actors (see [8]; Figure 1). During the original validation, children categorized each image according to the expressed emotion and rated the emotional intensity (high vs. low). For the current study, a total of 32 static images of child actors were selected to closely match the emotional expressions portrayed in the adult stimuli. These images were presented in a pseudo-randomized sequence to minimize repetition and order effects. Although a total of 32 stimuli (8 per emotion) may seem limited, this number was chosen based on prior studies using similar paradigms with young children [8,9,29]. Pilot testing confirmed this was sufficient to maintain attention and reduce fatigue while capturing consistent gaze patterns. Both children and parents completed the same experiment described above.

### 2.5. Apparatus

Eye-Tracking Apparatus: The experiment was conducted using the Tobii Pro Nano eye-tracker with Tobii Pro Studio software version 3.4.3 (Tobii Technology, Inc., Washington, DC, USA), designed to measure real-time attention allocation during an emotion-processing task. The Tobii Pro Nano is a bright-pupil eye-tracking system that uses a high-resolution camera and wide field of view to track eye movements at a sampling rate of 60 Hz. It employs two near-infrared diodes to illuminate the participant’s eyes, generating reflection patterns on the corneas. The high-resolution camera captures these reflection patterns along with the participant’s position relative to the screen. Attention allocation is determined by calculating the pupil’s position in the video signal throughout the recording. Pictures were displayed on a 22-inch computer screen with a maximum resolution of 1680 × 1050 pixels. Each facial stimulus measured approximately 12 cm × 8 cm, subtending a visual angle of approximately 14.3° × 18.6° at a viewing distance of 60 cm. All stimuli were presented under standardized lighting conditions. Images from the Radboud Faces Database were taken against a neutral gray background, and luminance and contrast were consistent across all stimuli. Trials with missing or incomplete eye-tracking data (e.g., due to blinks, head movement, or calibration failure) were excluded on a per-trial basis. If more than 30% of gaze data was missing for a participant across trials, the participant was excluded from final analyses. A total of 16 dyads were subsequently excluded due to the aforementioned criteria (see also Section 2.1). As part of the standard procedure, a 5-point calibration system was used to map each participant’s pupil position to corresponding gaze points on the screen.

Calibration was conducted prior to the facial emotion processing task to ensure the accurate recording of eye gaze. Once calibration was successfully completed, participants proceeded with the task. The exact timing of the events, such as the presentation of the visual affective stimuli and the tracking of eye gaze behavior in real-time, were developed on the Tobii Pro Studio 3.4.3. following the manufacturer’s standard practices and recommendations (Tobii Studio User’s Manual 3.4.3). To test the study’s hypotheses, Areas of Interest (AOIs) were defined around the eye and mouth regions. These were delineated using rectangular boundaries aligned with the upper, lower, left, and right edges of the eyes and mouth (see Figure 1), ensuring uniform AOI dimensions across all facial emotion stimuli used in the experiment. For gaze data processing, the primary outcome measures were the average fixation durations within each AOI (eyes and mouth), calculated separately for each emotional expression (anger, fear, sadness, and happiness). Eye-tracking recordings were processed offline using Tobii Pro Lab 24.21 software and Eye Tracker Manager 2.6.1., following the manufacturer’s standard guidelines.

### 2.6. Experimental Procedure

Upon arrival at the lab, families were welcomed by a researcher who explained the consent form and addressed any questions regarding the study. Children also provided assent before participating in the experiment. Once written consent was obtained, both parents and children were briefed on the procedure. Children were then escorted to the main lab area, where they were seated on a height-adjustable chair in front of a desktop computer in a well-lit room. The setup was adjusted to ensure the child could comfortably face the screen, allowing for optimal gaze tracking. The child’s head was positioned approximately 60 cm from the monitor, and they were instructed to avoid head movements or covering their face. A 5-point calibration was then conducted. After the child completed the task, the accompanying parent—who had been waiting in an adjacent room—underwent the same procedure. During calibration, participants were instructed to follow a green circle as it moved around the screen. This step was repeated until high-accuracy gaze calibration was achieved for both child and adult participants.

Stimuli were presented for three seconds. Each trial consisted of (1) a one-second fixation cross appearing in the center of the screen, and (2) a three-second presentation of the static facial expression. The task took approximately 5 min to be completed. After the completion of the experimental phase, families were informed in detail about the objectives of the study.

### 2.7. Data Analyses

All analyses were conducted in SPSS 29 (IBM Statistics, 2022, Armonk, NY, USA: IBM Corp). using standard and PROCESS macro models [30]. Variables used in moderation or mediation analyses were standardized (z-scores). Preliminary analyses included descriptive statistics, sex comparisons (*t*-tests), and zero-order correlations among child and parent SPS scores, empathy, emotional symptoms, and eye gaze indices. Following guidance in the literature suggesting the existence of a bifactor model of SPS for children with a general sensitivity factor and two specific factors (i.e., a combined LST-EOE and a separate AES factor; [14]), and with the aim to allow comparisons between the subscales of SPS between children and adults, the subscales of LST-EOE were summed into a total score for both children and adults. When assumptions of normality were violated for the independent variables, Spearman correlation analyses were used in place of Pearson correlations. Independent samples t-tests were conducted to examine gender differences in questionnaire scores, using the total scores from each measure. All questionnaire variables included in interaction analyses were treated as continuous and standardized using z-scores.

Hierarchical regressions were used to examine predictors of child eye gaze to emotional faces. Step 1 included parental eye gaze, child SPS subscales, empathy, and affective symptoms. Step 2 tested interaction terms (e.g., AES × cognitive empathy). The above analyses were conducted separately for parental-child eye gaze data per emotion and feature. Child sex was included as a covariate in the analyses. Significant interactions were visualized using an open-source interactive data visualization tool [31]. To aid interpretation, both multiple small plots—displaying separate plots for each level of the moderator—and marginal effects plots—illustrating regions of significance—were utilized.

Finally, a moderated mediation analysis was conducted using PROCESS Model 7 [30] to test: (a) whether child AES mediates the association between parental and child eye gaze, (b) whether child empathy moderates this mediation, (c) to run separate models for cognitive and affective empathy as moderators. The analysis employed a moderated mediation approach (Model 7 from [30]) to estimate the conditional indirect effect of parental eye gaze on child eye gaze, moderated by different facets of child empathy. The significance of both direct and indirect effects was assessed using 5000 bootstrap samples to generate bias-corrected 95% confidence intervals, with heteroscedasticity-consistent standard errors [30]. Child sex was included as a covariate in all models. Statistical significance was determined based on the bootstrapped confidence intervals.

## 3. Results

### 3.1. Descriptive Statistics

Table 1 presents the Means (M) and Standard Deviations (SD) for child and parent Sensory Processing Sensitivity (SPS), as well as children’s affective problems. Independent samples t-tests were conducted to examine potential differences in questionnaire scores based on child and parent sex. No significant gender-based differences were observed.

### 3.2. Behavioral Correlations

Correlation analyses showed a positive correlation between child total HSC score and parental HSP score (r = 0.375, *p* < 0.001), child anxiety traits (r = 0.377, *p* < 0.001), child affective (r = 0.385, *p* < 0.001) and cognitive empathy (r = 0.334, *p* < 0.001). The AES subscale of HSC was positively correlated with parental AES (r = 0.449, *p* < 0.001), child affective (r = 0.317, *p* < 0.001) and cognitive empathy (r = 0.500, *p* < 0.001). Children’s anxiety traits were also positively correlated with the parental total HSP score (r = 0.301, *p* < 0.001), and child depression rates (r = 0.625, *p* < 0.001; See Table 1).

### 3.3. Behavior by Eye Gaze Correlations

Correlation analyses showed negative correlations between child AES and parental total fixation duration towards angry eyes (r = −0.225, *p* = 0.012), angry mouth (r = −0.229, *p* = 0.011), fearful eyes (r = −0.238, *p* = 0.008), fearful mouth (r = −0.223, *p* = 0.013), happy eyes (r = −0.240, *p* = 0.007), happy mouth (r = −0.241, *p* = 0.007), sad eyes (r = −0.217, *p* = 0.015), and sad mouth (r = −0.211, *p* = 0.019). Parental SPS and the corresponding subscales, and child behavioral rates did not show any significant correlations with parental or child eye gaze patterns.

### 3.4. Behavior by Dyadic Eye Gaze Associations

Findings from Step 1 of the hierarchical regression analyses revealed that children’s eye gaze towards the angry eyes regions was significantly predicted by parental eye gaze towards the angry eyes regions (*p* = 0.013), child AES (*p* = 0.015), and child cognitive (*p* = 0.008) and affective empathy (*p* = 0.011). The model was significant in Step 1 at *p* = 0.042. Step 2 of the regression included the interaction terms child AES by Cognitive Empathy and child AES by Affective Empathy which did not show any additional significant effects of the interaction terms (model not significant *p* = 0.086; see also Table 2.). Subsequent hierarchical regression analyses at Step 1 were not found to be significant for all the remaining AOIs, i.e., angry mouth (*p* = 0.132), fearful eyes region (*p* = 0.123), fearful mouth (*p* = 0.193), happy eyes (*p* = 0.087), happy mouth (*p* = 0.113), sad eyes (*p* = 0.095), and sad mouth (*p* = 0.094).

### 3.5. Moderated Mediation Analyses

To test whether child Aesthetic Sensitivity (AES) mediated the relationship between parental and child eye gaze toward angry eyes, and whether this mediation was moderated by children’s empathy, we conducted a series of moderated mediation analyses using the PROCESS macro (Model 7; [30]).

**Figure 2:**
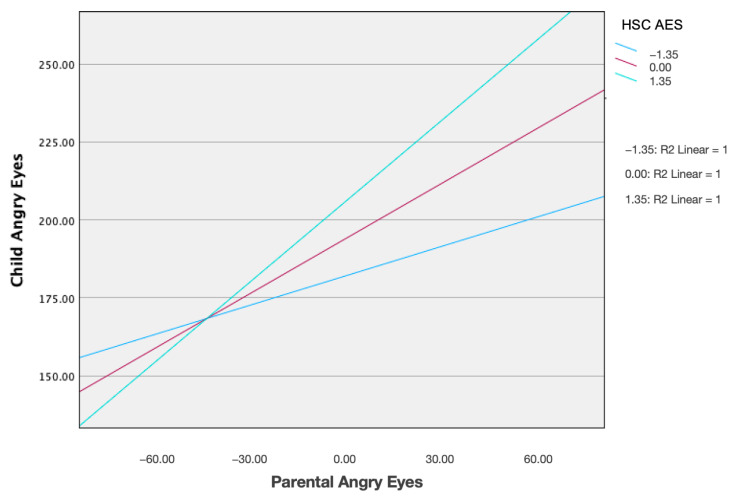
Depiction of the overall direct effect of child Aesthetic Sensitivity on the interaction between child-parent eye gaze when processing the angry eyes region.

**Figure 3:**
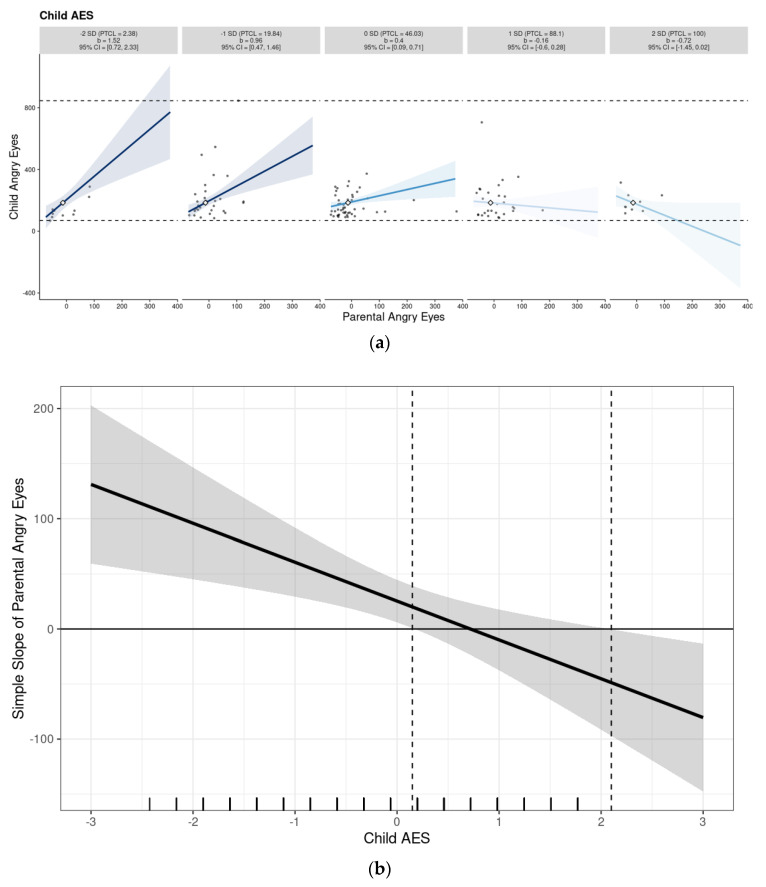
(**a**) Multiple small plots, which present an individual plot for each level of the moderator Child AES, and (**b**) marginal effect plot, which provide a visualization of the regions of significance. The simple slope of parental eye gaze on child eye gaze is significant and positive when child AES is 0.15 standard deviations away from the mean or further. A total of 46.03% of observations in child AES are within this region.

In the first model, we examined the mediating role of child AES in the association between parental fixation duration on angry eyes and the corresponding child gaze. The model accounted for 7.9% of the variance in child gaze behavior (R^2^ = 0.079, F (3, 120) = 3.47, *p* = 0.018). Although the overall indirect effect was modest, bootstrapped analyses revealed a significant conditional indirect effect at higher levels of AES (+1 SD), indicating that the association between parent and child gaze was stronger among children with elevated AES (see Figure 2 and Figure 3; Table 2 and Table 3). This finding suggests that children who are more aesthetically sensitive may be more influenced by their parent’s visual attention patterns when processing emotionally salient stimuli, specifically angry facial expressions.

In the second model, we explored whether empathy moderated the mediation effect of AES. Two separate moderation models were conducted, one for cognitive empathy and one for affective empathy. In both cases, the interaction terms between AES and empathy were non-significant (cognitive empathy: β = 0.02, t = 0.33, *p* > 0.05; affective empathy: β < 0.01, t = 0.68, *p* > 0.05), indicating no evidence for a moderated mediation effect. Although cognitive and affective empathy were each positively associated with children’s gaze behavior in preliminary analyses, they did not significantly alter the strength or direction of the mediation pathway.

Taken together, these analyses provide support for the mediating role of child AES in the relationship between parental and child eye gaze to angry eyes but do not support the hypothesis that this pathway is moderated by empathy. The findings underscore the unique contribution of aesthetic sensitivity in explaining how children may align with their parents’ attentional patterns during emotional face processing.

## 4. Discussion

The present study examined whether parental eye gaze to emotional facial expressions is associated with corresponding gaze behavior in children, and whether this association is statistically accounted for by child Aesthetic Sensitivity (AES), a subcomponent of Sensory Processing Sensitivity (SPS), and varies as a function of empathy. Using a dyadic eye-tracking paradigm, we found that children’s gaze toward angry eyes was significantly predicted by their parents’ gaze to the same emotional region. This association was statistically accounted for by child AES, suggesting that children with higher aesthetic sensitivity tend to show greater alignment with their parents’ emotional attention patterns. However, empathy—while independently predictive of gaze—did not significantly moderate the mediating pathway. These findings align with and extend existing work on intergenerational transmission of emotional processing (e.g., [20,21]). While previous studies have primarily focused on behavioral or clinical outcomes (e.g., [7,8,9]), this study is among the first to examine shared parent–child visual attention patterns to emotional stimuli in relation to trait-level child sensitivity. The finding that AES statistically accounted for the parent–child gaze association specifically for angry eyes indicates that emotionally charged or socially significant stimuli—such as expressions of anger—may be particularly salient for children high in aesthetic sensitivity. Contrary to our hypothesis, we did not find support for empathy as a moderator of this relationship. While cognitive and affective empathy were both associated with children’s gaze behavior in regression models, neither significantly altered the strength of the mediation pathway involving AES. One possibility is that empathy and AES reflect overlapping but distinct aspects of emotional reactivity and awareness, with AES capturing more automatic or perceptual elements of sensitivity, and empathy involving more cognitively mediated processes. Notably, cognitive empathy was associated with reduced gaze toward angry eyes, potentially indicating an avoidant strategy in children who intellectually recognize emotional content but do not engage visually. In contrast, affective empathy was associated with increased gaze, possibly reflecting heightened emotional engagement. This dissociation highlights the need to consider the nuanced roles of empathy subtypes in children’s responses to social-emotional stimuli.

Our findings also refine previous conceptualizations of SPS. While AES was significantly implicated in predicting gaze behavior and mediating parent–child alignment, the EOE-LST subscales were not. This aligns with the literature linking AES to behavioral activation (BAS), positive affect, and sensitivity to emotional nuances [16,17]. That this effect was strongest in response to angry expressions—traditionally considered negative—may seem unexpected. However, anger can serve as a socially relevant signal requiring attention and resolution, particularly within close interpersonal contexts. Children high in AES may be especially tuned to such cues due to their heightened perceptual awareness and social receptivity.

These results provide partial support for the study’s hypotheses. Hypothesis 1—that parent and child eye gaze would align—was supported for angry eyes. Hypothesis 2, proposing a mediating role of SPS, was supported for AES but not other subscales. Hypothesis 3, which predicted that AES would relate to positive stimuli and EOE-LST to negative stimuli, was not supported, as gaze effects were specific to AES and angry eyes. Hypothesis 4, concerning the moderating role of empathy, was not supported, though empathy was independently associated with gaze behavior.

Several limitations should be noted. First, the sample size, while sufficient for the planned analyses, limits generalizability and may reduce power for detecting interaction effects. Second, the cross-sectional design precludes causal inference and limits our ability to assess developmental change. Longitudinal designs are needed to examine how these attentional patterns and trait interactions unfold over time. Third, reliance on parent-report measures of child traits may introduce shared method variance or bias, particularly in SPS and empathy ratings. Future studies should incorporate child self-report, behavioral assessments, or physiological indices to triangulate findings. Additionally, the study focused only on one parent—typically the mother—which limits conclusions about paternal influence or the broader family dynamic. Fourth, the use of static facial stimuli may not fully capture the dynamics of real-world emotion processing. Incorporating dynamic or interactive emotional tasks could enhance ecological validity and provide deeper insight into attention and regulation in social contexts. Moreover, as the sample consisted solely of Greek Cypriot parent–child dyads, the findings should be interpreted within this cultural context. Future research should replicate this design across diverse cultural settings to assess the generalizability of shared gaze patterns and trait influences. Finally, while AES emerged as a key mechanism, we did not explore potential contextual moderators, such as parenting style, family stress, or attachment security. These may interact with child traits to shape attentional outcomes and should be incorporated in future research.

## 5. Conclusions

This study contributes to the growing literature on Environmental Sensitivity by highlighting Aesthetic Sensitivity as a mechanism through which children align their attentional responses to emotional stimuli with their parents. While empathy was not found to be associated with this relationship, its association with gaze behavior suggests a complex interplay between trait-level sensitivity and social-cognitive functioning. These findings offer preliminary evidence for trait-dependent intergenerational patterns in emotional attention, with implications for understanding how children perceive and internalize emotionally relevant cues within family contexts. Further investigation of these processes may inform prevention and intervention strategies that consider child temperament in the development of socio-emotional competencies.

## Figures and Tables

**Table 1:** Descriptive Statistics and Correlations among child SPS levels and behavioral traits and parental SPS.

Measure	1	2	3	4	5	6	7	8	9	10
1. HSC										
2. HSC-EOE/LST	0.881 **									
3. HSC-AES	0.765 **	0.369 **								
4. HSP	0.375 **	0.340 **	0.272 **							
5. HSP-AES	0.431 **	0.292 **	0.602 **	0.602 **						
6. HSP-EOE/LST	0.289 **	0.272 **	0.787 **	0.331 **	0.331 **					
7. Anxiety	0.380 **	0.432 **	0.302 **	0.132	0.185 *	0.185 *				
8. Depression	0.347 **	0.402 **	0.243 **	0.188 *	0.094	0.294	545 **			
9. Cog Emp	0.334 **	0.114	0.062	0.253 **	0.011	−0.029	−0.029	−0.071		
10. Aff Emp	0.385 **	0.322 **	0.083	0.121	0.079	0.109	0.109	0.118	0.622 **	
Descriptives										
Mean	3.64	2.77	5.06	4.34	5.31	3.91	1.60	1.18	3.80	3.67
SD	1.00	1.12	1.34	1.00	0.95	1.55	0.50	0.26	0.57	0.63

Note: ** Correlation is significant at the 0.01 level (2-tailed). * Correlation is significant at the 0.05 level (2-tailed). HSC = Highly Sensitive Child; HSP = Highly Sensitive Person; EOE = Ease of Excitation; AES = Aesthetic Sensitivity; LST = Low Sensory Threshold; Cog Emp = Cognitive Empathy; Aff Emp = Affective Empathy.

**Table 2:** Results of regression analysis with child eye gaze towards the angry eyes region as the outcome.

Variable	Child Eye Gaze: Angry Eyes						
	B	SE B	β	t	r^2^	r^2^ Adjusted	*p*
Model 1					0.398	0.158	0.042
Child Gender	28.1	20.6	0.12	1.26			
Depression	−34.2	47.4	−0.07	−0.72			
Anxiety	−7.11	25.0	−0.32	−0.28			
Child SPS AES	24.8	10.0	0.29	2.47 *			
Child SPS EOE	−4.80	11.2	−0.48	−0.42			
Aff Emp	54.7	21.2	0.30	2.57 *			
Cog Emp	−68.4	25.4	−0.34	−2.69 **			
Parental Total SPS	−7.94	20.6	−0.70	−0.38			
Parental SPS-AES	−10.85	14.8	−0.09	−0.72			
Parental SPS-EOE/LST	4.83	11.0	0.06	0.43			
Parental Eye Gaze	0.42	0.16	0.23	2.51 *			
Model 2					0.401	0.161	0.086
Child Gender	28.0	20.7	0.12	1.35			
Depression	−33.4	47.8	−0.07	−0.69			
Anxiety	−8.26	25.2	−0.03	−0.32			
Child SPS AES	24.6	10.1	0.29	2.43 *			
Child SPS EOE	−4.71	11.30	−0.04	−0.41			
Affective Empathy	54.1	22.22	0.30	2.43 *			
Cognitive Empathy	−69.5	25.92	−0.35	−2.68 **			
Parental Total SPS	−5.14	22.22	0.30	2.43			
Parental SPS-AES	−13.19	15.93	−0.11	−0.82			
Parental SPS-EOE/LST	4.58	11.32	0.06	0.40			
Parental Eye Gaze	0.46	0.17	0.23	2.50 *			
Child AES × Cog Emp	−0.11	15.50	−0.00	−0.00			
Child AES × Aff Emp	−5.02	13.85	−0.05	−0.36			

Note: All measures, except parental SPS and eye gaze, refer to children’s characteristics and behaviors. Cog Emp = Cognitive Empathy; Aff Emp = Affective Empathy. * *p* < 0.02; ** *p* < 0.01.

**Table 3:** Conditional effects of the focal predictor of the child–parent relationship on eye gaze at values of the children’s aesthetic sensitivity as the moderator.

HSC AES	β	SE	t	*p*	LLCI	ULCI
−1.346	0.310	0.167	1.864	0.065	−0.019	0.640
0.000	0.581	0.189	3.081	0.003	0.208	0.955
1.346	0.852	0.289	2.949	0.004	0.280	1.424

Regression coefficient = β; standard error = SE; *t*-test values = t; LLCI = Lower Limit Confidence Intervals; ULCI = Upper Limit Confidence Intervals.

## Data Availability

The datasets generated and/or analyzed during the current study are available from the corresponding author upon reasonable request due to privacy.
